# The fate and function of vision during saccades

**DOI:** 10.16910/jemr.12.7.4

**Published:** 2019-11-25

**Authors:** Martin Rolfs

**Affiliations:** Humboldt University of Berlin

**Keywords:** vision, saccade, attention

## Abstract

Keynote at the 20th European Conference on Eye Movement Research (ECEM) in Alicante, 20.8.2019,

(Video stream not punlished on internet)

